# Beyond the Absent Father Myth: How Structural Violence Through ACEs and APIs Impacts the Father-Child Relationship

**DOI:** 10.1093/geroni/igaf122.3293

**Published:** 2025-12-31

**Authors:** Milan Riddick, Muriel Taks Calle, Paris Adkins-Jackson

**Affiliations:** Columbia University Mailman School of Public Health, New York, New York, United States; Columbia University Mailman School of Public Health, New York, New York, United States; Mailman School of Public Health, Columbia University, New York, New York, United States

## Abstract

Men racialized as Black are often stereotyped as “absent fathers”, a characterization that overlooks their presence and absolves the role of structural racism in their lives. Adverse Childhood Experiences (ACEs) and Adverse Police Interactions (APIs) likely influence fathers’ relationships with their children. We analyzed a purposive sample of fathers racialized as Black from the Health and Retirement Study (aged 51+, N = 147). ACEs were measured using a sum of 10 self-reported adverse experiences occurring before age 18, while APIs were indicated by having had trouble with the police before age 16, experiencing unfair police encounters, and/or imprisonment for at least three days. Dependent variables included Contact — the frequency of interactions between fathers and children; Positive Relation —measuring the extent to which children understand, support, and connect with their father; and Negative Relation —measuring how frequently children make demands of the father, criticize him, disappoint him, or make him feel unnerved. Regression analyses revealed that APIs are associated with decreased contact between fathers and their children (β = -0.920*; 95% CI [-1.93, 0.09]) and increased negative relation (β = 1.022*; 95% CI [-0.06, 1.13]). ACEs are associated with a decrease in positive relation (β = -0.472***; 95% CI [-0.538, -0.130]) and an increase in negative relation (β = 0.249**; 95% CI [0.04, 0.43]). These findings suggest both ACEs and APIs undermine fathers’ abilities to maintain healthy relationships with their children. There is a need to shift from harmful stereotypes to addressing structural violence that shape these integrational dynamics. *p< 0.1, **p< 0.05, ***p< 0.01

